# A comparison among employees in Germany and Denmark of associations between quality of leadership and subsequent 5-year development of mental distress

**DOI:** 10.1038/s41598-025-92650-0

**Published:** 2025-03-06

**Authors:** Hermann Burr, Norbert Kersten, Kathrine Sørensen, Jeppe K. Sørensen, Louise Dalsager, Ida E. H. Madsen, Ina Schöllgen, Angelo d’Errico, Uwe Rose, Reiner Rugulies

**Affiliations:** 1 Department of Work and Health, Federal Institute for Occupational Safety and Health (BAuA), 10317 Berlin, Germany; 2 National Research Centre for the Working Environment (NRCWE), 2100 Copenhagen Ø, Denmark; 3 Department of Psychology, University of Copenhagen, 1353 Copenhagen K, Denmark; 4 National Institute of Public Health, University of Southern Denmark, 1455 Copenhagen K, Denmark; 5 Department of Epidemiology, Local Health Unit TO 3, 10095 Grugliasco Turin, Italy; 6 Section of Epidemiology, Department of Public Health, University of Copenhagen, 1353 Copenhagen K, Denmark

**Keywords:** Cross-country studies, Psychosocial working conditions, Mental health, Risk factors, Depression

## Abstract

We hypothesised that the association between quality of leadership and mental distress would be stronger in Germany than in Denmark. The two countries differ in terms of working life cultures and labour markets, factors possibly mitigating the association of quality of leadership with mental distress. Data were based on the German Study on Mental Health at Work (2012–2017) and the Danish Work Environment Cohort Study (2000–2005 and 2005–2010), comprising 9,613 observations. A two-part model approach was employed, combining logistic and generalized linear regression models, with mental distress at follow-up as outcome variable. Heterogeneity of the country-specific estimates was assessed using a Cochran Q test. Among all participating employees, the logistic regression showed that higher quality of leadership was associated with lower mental distress (> 0) in both Germany and Denmark. This association was stronger in Germany, with the country difference being statistically significant (*p* = 0.036). Among employees with mental distress at follow-up, the generalized linear models revealed that quality of leadership was associated with decreased mental distress in Denmark, but not in Germany, with the country difference also being significant (*p* = 0.014). The inconsistent results did not support our hypothesis of a stronger association between quality of leadership and mental health in Germany. All results taken into consideration, the findings suggest that poor quality of leadership is associated with the development of mental distress in both countries. Further research is needed to examine the association between quality of leadership and mental health in other countries.

**Supplementary Information:**

The online version contains supplementary material available at 10.1038/s41598-025-92650-0.

## Introduction

There is an increasing interest in the role of psychosocial work environment factors in the aetiology of mental distress^1^. In this context, the notion of mental distress comprises not only depression and depressive symptoms but also states of poor mental well-being, such as perceived stress^2^. Analyses of work and mental distress in prospective cohort studies are largely confined to a few dimensions of the psychosocial work environment, namely job strain, effort-reward imbalance (ERI), social support, workplace bullying and long working hours^1,3,4^.

In a working life that has become more and more complex, research on work and health has increasingly focused on the role of leadership and cooperation, which goes beyond the above mentioned dimensions^5^. Leadership can be defined as the capacity to help others in fulfilling a goal by means of processes of influence^6^. Thus, quality of leadership can be understood as comprising various aspects of a leader’s behaviour towards their subordinates, such as providing opportunities for development, solving conflicts, caring for the employees’ well-being, recognizing their contributions, and effectively carrying out these processes^7,8^. Important aspects of leadership are trust, justice, social support and cooperation^9^. Among a larger range of psychosocial working conditions, these aspects have been found to be the most strongly associated with quality of leadership^10,11^. Leadership quality rated low in terms of justice and support has been linked to depressive symptoms and other health complaints^12,13^.

Therefore, we expect low quality of leadership to be associated with mental distress. Quality of leadership has been measured either through various items (e.g. if the immediate superior ensures development opportunities, or if the employee experiences recognition)^8,10^, or by combining subscales that measure different beneficial leadership styles—such as empowering and fair leadership—into a global measure^14^. Another approach is to focus on specific beneficial leadership styles, such as transformational, empowering or fair leadership, without combining these into one single global measure^15–17^.

To our knowledge, four longitudinal studies have assessed the association between quality of leadership and mental distress so far^14,18–20^. Two of these studies were based on national samples from Sweden and Denmark^19,20^, one study focused on a diverse range of Norwegian workplaces^14^, and one study examined a specific Danish industry^18^. Three of these studies reported a statistically significant association between baseline indicators of leadership and mental distress at follow-up^14,18,20^, and one reported no association^19^. Four Scandinavian studies investigated prospective associations between specific beneficial leadership styles and mental distress —two focused on a wide range of workplaces in Norway, and two on specific industries in Denmark and Sweden^14,21–23^. Regarding empowering leadership, two studies found statistically significant associations^14,21^. For fair leadership, the same two studies reported mixed findings: one found a significant association, while the other did not^14,21^. Similarly, for transformational leadership two other studies reported mixed findings, with one showing a significant and the other a non-significant association^22,23^. The outcomes of some of the studies mentioned above included treatment or hospitalization for depression or anxiety^19,20^, or depressive symptoms^18,20^ or mental health symptoms^14^ above a clinical cut-off point. Thus, these studies were limited to clinical mental health conditions and did not examine the full spectrum of mental distress.

All aforementioned studies on leadership and mental distress were based on Scandinavian cohorts, which is a concern, as the associations between psychosocial work environment factors and mental health may depend on national contexts^24–26^. From an international comparative perspective, Scandinavian countries differ from other countries in terms of working life cultures and the labour market^27–30^. These factors may affect quality of leadership and potentially mitigate its association with mental distress. Furthermore, in the Scandinavian context leadership is often more participative and team oriented^28,29^, while the labour market is characterized by higher union membership density and more active labour market policies and lower income inequality^27,30^. In conservative welfare states, leadership values are less participative and team oriented, union membership density is lower, labour market policies are less active, and income inequality is higher^27–30^. Therefore, it can be assumed that associations between quality of leadership and mental distress are stronger in conservative welfare states than in Scandinavian, as aspects of working life cultures and the labour market in the latter may buffer these associations.

In the present study, we examined data on quality of leadership and mental distress from two cohort studies in Denmark and Germany^31,32^, representing the above mentioned Scandinavian and conservative welfare states, respectively^29,30^. Specifically, we conducted a cross-national prospective study to investigate whether the longitudinal associations between quality of leadership and mental distress differ between Germany and Denmark. We hypothesized that the association would be stronger in Germany than in Denmark.

## Methods

### Study population

We used data from two cohort studies with a 5-year follow-up, that is, the German Study on Mental Health at Work (S-MGA) and the Danish Work Environment Cohort Study (DWECS). The S-MGA study included a baseline measurement in 2012 and a follow-up in 2017, while the DWECS study included baseline measurements in 2000 and 2005, with follow-ups in 2005 and 2010.

### German employee cohort

**Fig. 1:**
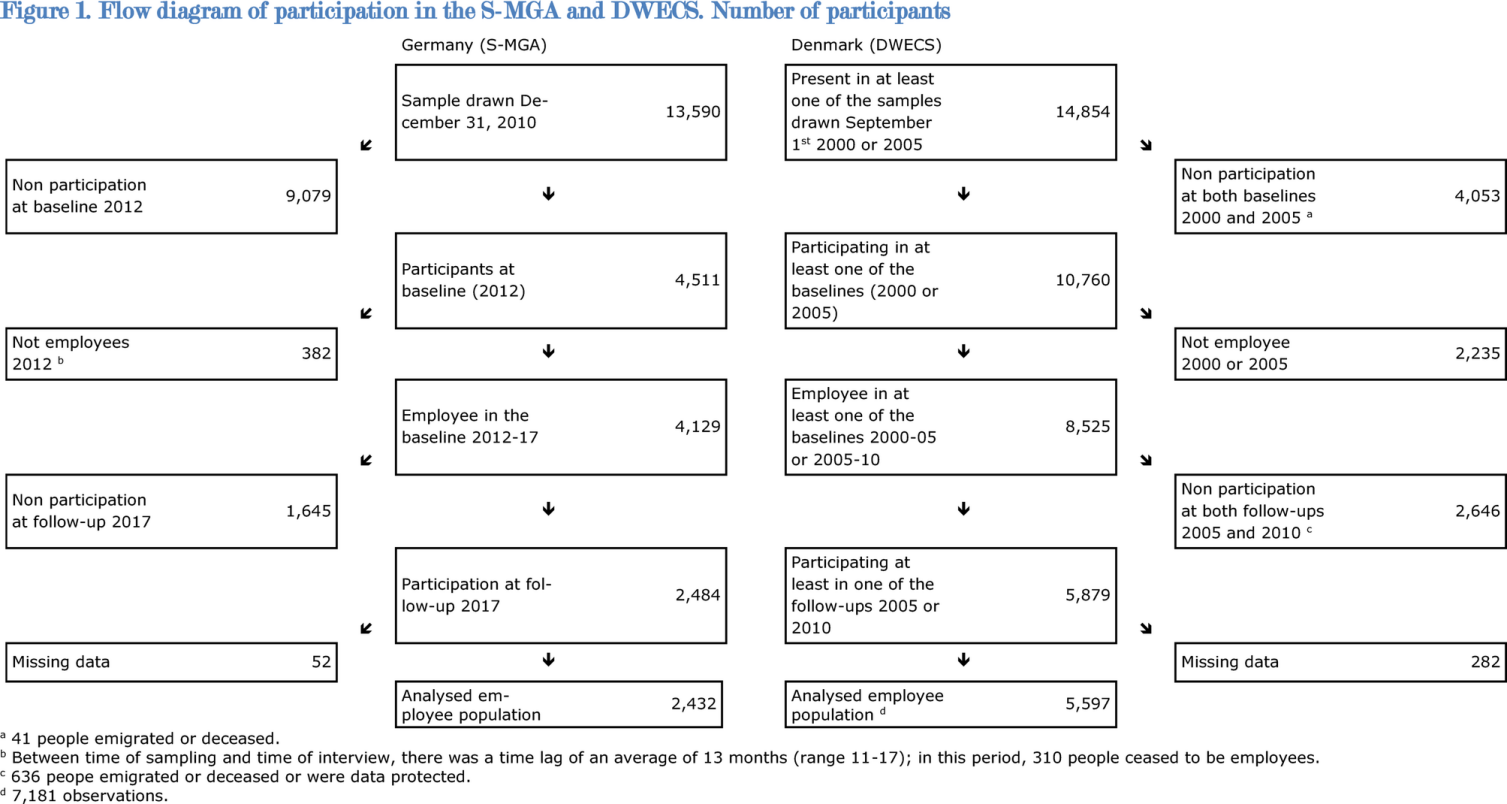
Flow diagram of participation in the S-MGA and DWECS. Number of participants

The S-MGA is a nation-wide employee cohort study with a baseline survey in 2012 and a follow-up in 2017, with a baseline response of 33% and a follow-up response of 59% (Fig. 1)^32^. At baseline, the target population consisted of all individuals employed in Germany on December 31st 2010, born in 1951–1980^32^. The study population was randomly sampled from the register of Integrated Employment Biographies (IEB) of the German Federal Employment Agency at the Institute for Employment Research (IAB). This register covers 80% of all employees within this age range, excluding the 20% who were civil servants, self-employed workers and freelancers. The analysed cohort comprised 2,484 people who were employees at baseline, took part in the follow-up, and had non-missing information on all variables included in this study (Fig. 1). All respondents took part in computer-assisted personal interviews at their home address – both at baseline and at follow-up^32^.

### Danish employee cohort

We extracted the 2000–2005 and 2005–2010 cohorts from DWECS, which had a responses of 72% and 67% respectively at baseline and a responses of 72% and 67% respectively at follow-up (Table 3). DWECS was an open population-based cohort study investigating work and health among the Danish workforce through repeated questionnaire assessments conducted every five years^33,34^. Thus, a DWECS participant could have been included in one or both 5-year cohorts. The cohort comprised 7181 observations from 5,597 people who were employed at baseline, took part in at least one follow-up, and had non-missing information on all key variables (Fig. 1). Respondents either took part in computer assisted telephone interviews or filled out paper questionnaires. The change in mode of data collection from telephone interviews was partly introduced in 2005 and was fully implemented in 2010. Among the 7181 observations, 790 were collected through telephone interviews at both baseline and follow-up, 3572 were collected through questionnaires at both baseline and follow-up, and 2819 observations were via telephone interviews at baseline and via questionnaire at follow-up.

**Table 1:** Participation in interviews at follow-up and in the cohort by gender and age at baseline.

	German cohort: 2012–2017 ^(a)^			Danish cohort: 2000–2005 ^(b)^			Danish cohort: 2005–2010 ^b)^		
	Baseline response ^(c)^; %	Follow-up response among baseline employees ^(d)^, %	Cohort fraction of the drawn sample ^(e)^, %	Baseline response ^(f)^; %	Follow-up response among baseline employees ^(g)^, %	Cohort fraction of the drawn sample ^(h)^, %	Baseline response ^(i)^; %	Follow-up response among baseline employees ^(j)^, %	Cohort fraction of the drawn sample ^(k)^, %
Gender									
Male	33%	58%	19%	73%	68%	50%	61%	64%	39%
Female	33%	60%	20%	78%	73%	57%	70%	70%	48%
Age									
55–60	39%	59%	23%	73%	78%	57%	68%	70%	48%
49–54	35%	61%	21%	75%	74%	55%	69%	71%	49%
43–48	33%	60%	20%	76%	72%	55%	67%	70%	46%
37–42	32%	58%	19%	77%	70%	54%	63%	66%	41%
31–36	27%	54%	14%	77%	64%	50%	60%	59%	35%
Total	33%	59%	19%	76%	72%	54%	65%	67%	44%

^(a)^ Based on published baseline attrition analyses^32^ and on available data in the SMGA data set. ^(b)^ Based on available data in the DWECS dataset. ^(c)^ Fraction being interviewed at baseline (4,511) of the drawn sample (13,590), see Fig. 1. ^(d)^ Fraction being interviewed at follow-up and with non-missing information (2,432) of the employees interviewed at baseline (4,129), see Fig. 1. ^(e)^ Fraction in the analysed cohort of the drawn sample (estimated by multiplying the fraction of the baseline response with the fraction of follow-up response among baseline employees). ^(f)^ Fraction being interviewed at baseline (5,409) of the drawn sample (7,152). ^(g)^ Fraction being interviewed at follow-up and with non-missing information (2,834) of the employees interviewed at baseline (4,072 − 66 died or emigrated during follow-up), see Fig. 1^(h)^ Fraction in the analysed cohort of the drawn sample (estimated by multiplying the fraction of the baseline response with the fraction of follow-up response among baseline employees). ^(i)^ Fraction being interviewed at baseline (8583) of the drawn sample (13,168). ^(j)^ Fraction being interviewed at follow-up and with non-missing information (4,347) of the employees interviewed at baseline (6,801 − 329 died or emigrated during follow-up). ^(k)^ Fraction in the analysed cohort of the drawn sample (estimated by multiplying the fraction of the baseline response with the fraction of follow-up response among baseline employees).

### The composition of the analysed cohorts

Attrition was not related to gender in the German part of the cohort, while in the Danish part attrition was lower among men (Table 1). In both cohorts, participation was lower among the youngest workers. Compared to occupational level, quality of leadership and mental distress at baseline, participation at follow-up was lower among unskilled workers. However, the patterns of participation were less clear in relation to quality of leadership and mental distress (Table 2). As for quality of leadership, cohort differences in participation were negligible in the German cohort and in the Danish 2005-10 cohort, whereas in the Danish 2000-05 cohort, follow-up participation was lower among those with higher levels of quality of leadership at baseline. Regarding mental distress, differences in participation were negligible in the German cohort. However, in the Danish part of the study, follow-up participation was lower among those participants without mental distress, especially in the 2005-10 cohort.

**Table 2:** Participation in interviews at follow-up by baseline occupational level, quality of leadership and mental distress.

	German cohort	Danish cohorts	
	2012-17	2000-05	2005-10
Occupational level^(a)^			
Professionals, Managers	66%	76%	70%
Semiprofessionals	63%	76%	72%
Skilled workers	55%	70%	64%
Unskilled workers	50%	63%	62%
Quality of leadership^(b)^			
< 1	59%	75%	70%
1-<2	60%	73%	69%
2-<3	59%	73%	71%
3–4	57%	69%	68%
Mental distress^(c)^			
0	58%	68%	58%
10	59%	71%	67%
20–30	61%	73%	71%
=>40	57%	73%	68%
Total	59%	71%	70%

^(a)^ Occupational level was largely based on the four category skill level International Standard Classification of Education (ISCED), see method Sects^42,43^. ISCED uses a categorization of the International Standard Classification of Occupations (ISCO)^42^.

^(b)^Scale ranging from 0 to 4 from the Copenhagen Psychosocial Questionnaire (COPSOQ)^38,39^.

^(c)^Two item Mental Health scale (MH-2) from the 12 item Short Form Health Survey (SF-12) going from 0 to 100, reversed so that 0 reflects lowest mental distress and 100 highest^35,36^.

**Table 3:** Description of the analysed population at baseline.

	Employees in Germany							Employees in Denmark						
	N	%	Mean	Std. dev.	Skew-ness.	α ^a^	IICR ^b^	N, observations	%	Mean	Std. dev.	Skewness	α ^a^	IICR ^b^
Gender														
Men	1,189	49						3,273	46					
Women	1,243	51						3,908	54					
Age, baseline (31–60)			46.9	7.6	-0.2					45.3	8.1	-0.0		
31–40 years	548	23						2,332	32					
41–50 years	1,028	42						2,623	37					
51–60 years	856	35						2,226	31					
Occupational level, baseline														
Unskilled workers (ISCO group 9) ^c^	137	6						655	9					
Skilled workers (ISCO group 4–8) ^c^	1,022	42						2,744	38					
Semi-professionals (ISCO group 3) ^c^	682	28						1,891	26					
Professions/managers (ISCO group 1,2) ^c^	591	24						1,891	26					
Quality of leadership, baseline (0–4)			2.3	0.9	-0.4	0.84	0.52;0.65			2.3	0.8	-0.4	0.87	0.56;0.68
Influence at work, baseline (0–4)			1.7	1.0	0.2	0.71	0.32;0.43			2.1	1.0	-0.1	0.77	0.41;0.55
Mental distress, baseline (0-100)			36.9	20.1	0.3	0.73	0.58 ^d^			21.8	16.6	1.2	0.61	0.44 ^d^
Total	2,432	100						7,181	100					

^a^ Cronbach’s α.

^b^ Inter item correlation range.

^c^ Occupational level was largely based on the four category skill level International Standard Classification of Education (ISCED), see method Sects^42,43^. ISCED uses a categorization of the International Standard Classification of Occupations (ISCO)^42^.

^d^. As the scale was based on only two items, there was only one inter item correlation.

9,613 observations among 8,029 employees.

In the baseline of both analysed cohorts, men and women were equally represented, with slightly more women in the Danish than the German cohort (54% vs. 51%, see Table 3). The age group 41–50 years was overrepresented in both cohorts, more so in the German cohort (42%) than in the Danish cohort (38%); the age group 31–40 years was underrepresented in the German cohort (23%).

## Variables

### Mental distress

Mental distress was measured with a scale by means of the following two mental health (MH-2) items from the 12 Item Short Form Health Survey (SF-12)^35–37^: “How much of the time during the past 4 weeks - have you felt downhearted and blue?’’ and ‘‘- have you felt calm and peaceful?’’ (reverse scored). In the S-MGA study, these two items were answered using five response options: “Always” (100), “Often” (75), “Sometimes” (50), “Almost never” (25) and “Never” (0). In the DWECS study, six response options were used: ‘‘All of the time’’ (100), ‘‘Most of the time’’ (80), ‘‘A good bit of the time’’ (60), ‘‘Some of the time’’ (40), ‘‘A little of the time’’ (20), and ‘‘None of the time’’ (0). The latter were the original response options from the SF-12. Cronbach α was 0.73 in the German sample, reflecting a Pearson item intercorrelation of 0.579, and 0.61 in the Danish sample, reflecting a Pearson item intercorrelation of 0.452 (Table 3). Among Danish participants, Cronbach αs were similar regardless of the of the mode of data collection (see Supplementary online Table 1). The distribution of the mental distress score was left-skewed in both studies, with a stronger skew observed in the Danish sample compared to the German sample (skewness, see Table 3). This is reflected by a higher mean level of mental distress in the German sample as compared to the Danish sample. The scale had a mean of 36.9 in the German baseline and a mean of 21.8 in the Danish baseline, indicating relatively low levels of mental distress in both. The pattern regarding skewness and difference of mean scores between the German and Danish baselines was the same regardless of the mode of data collection among Danish participants (see Supplementary online Table 1).

In a logistic regression, the mental distress scale was dichotomized due to its distribution (see the Statistical analyses subsection below), so that 0 indicated ‘No distress’ (scale score equal to 0) and 1 indicated ‘Distress’ (scale scores from > 0 to 4).

In the Danish baseline, all items of the mental health (MH-5) instrument from SF-36 were available^36,37^. We calculated a mental distress scale scoring the 5 response options as described above. We used this scale for a sensitivity analysis comparing this 5-item measure with the 2-item measure described above as independent variables.

### Quality of leadership

Quality of leadership was measured with four items from the Copenhagen Psychosocial Questionnaire (COPSOQ)^38,39^: ‘To what extent would you say that your immediate superior – makes sure that the individual member of staff has good development opportunities?’, ’– gives high priority to job satisfaction?’, ’ – is good at work planning?’ ’ – is good at solving conflicts?’. Response options and corresponding scales values were: ‘To a very large extent’ (4), ‘To a large extent’ (3), ‘Somewhat’ (2), ‘To a small extent’ (1), ‘To a very small extent’ (0). Scale scores were calculated as a mean of the component items, if at least half of them were non missing^38,39^. Cronbach α was 0.84 in the German baseline and 0.87 in the Danish baseline (Table 3). The scale had a mean of 2.3 in both baselines (Table 3).

### Covariates

We included gender, age, occupational level and influence at work, all measured at baseline, as potential confounders; in the Danish cohort, we additionally adjusted for year of data collection at baseline and mode of data collection at baseline and follow-up. Age was treated as both a linear and a quadratic term due to the non-linear relationship between age and mental distress^40,41^.

Occupational level was largely based on the four-category skill level classification from the International Standard Classification of Education (ISCED)^42,43^. ISCED is based on a categorization of the International Standard Classification of Occupations (ISCO)^42^. ISCO 2008 and ISCO 1988 were used in the S-MGA and DWECS studies, respectively. In both baselines, self-reported information on occupational level were subsequently coded based on ISCO. We collapsed the main ISCO group 2 into the category ‘Professionals/managers’; group 3 formed the category ‘Semi-professionals’; groups 4–8 were collapsed in the category ‘Skilled workers’; group 9 formed the category ‘Unskilled workers’. The ISCED skill level classification does not include the skill level of managers (ISCO main group 1). In accordance with classifications of socioeconomic position^44^, we then classified managers in the same group as professionals.

Mode of data collection had the following categories^33^: (i) interview at baseline and follow up, (ii) interview at baseline and questionnaire at follow up, and (iii) questionnaire at baseline and follow up. All three modes were present in the DWECS study, while only the first one was used in the S-MGA study.

Influence at work (i.e. decision authority) was a scale calculated as the mean of four items (Kristensen et al. 2005; Nübling et al. 2006): “Can you influence the amount of work assigned to you?”, “Do you have any influence on what you do at work?”, “Do you have a large degree of influence concerning your work?”, “Do you have a say in choosing who you work with?” ’. Response options and corresponding scale values were: ‘To a very large extent’ (4), ‘To a large extent’ (3), ‘Somewhat’ (2), ‘To a small extent’ (1), ‘To a very small extent’ (0), which were combined into a scale with values ranging from 0 to 4. Scale scores were calculated if at least half of items were non missing. Cronbach α was 0.71 in the German baseline and 0.77 in the Danish baseline (Table 3). The scale had a mean of 1.7 in the German baseline, whereas the mean was 2.1 in the Danish baseline (Table 3).

### Correlations between the independent variables

We investigated to what extent the baseline independent variables were correlated.

In the German baseline of the cohort, the highest Pearson intercorrelation was between influence at work and occupational level (0.26, p = < 0.001), the second largest between quality of leadership and mental distress (-0.24, p = < 0.001), the third largest correlation was between age and occupational level (-0.05, *p* = 0.008). The remaining intercorrelations ranged from 0.04 (*p* = 0.079; between age and mental distress) to -0.007 (*p* = 0.749; regarding age and quality of leadership). Regarding gender, influence at work (mean m: 1.83 vs. w. 1.56, p = < 0.001) was higher among men than among women, whereas quality of leadership (mean w. 2.31 vs. m. 2.22, p = < 0.023) and mental distress (mean w. 39.1 vs. m. 34.6, p = < 0.001) was higher among women). Age (mean m: 46.7 vs. w. 47.1, *p* = 0.126) and occupational level (mean w. 2.7 vs. m. 2.7, *p* = 0.662) did not differ significantly.

In the Danish baseline of the cohort, the highest Pearson intercorrelation was between influence at work and occupational level (0.29, p = < 0.001), the second highest was between quality of leadership and mental distress (-0.23, p = < 0.001), the third largest correlation was between quality of leadership and occupational level (-0.09, p = < 0.001). The remaining intercorrelations ranged from 0.05 (p = < 0.001; between age and mental distress) to -0.002 (*p* = 0.035; between age and quality of leadership). Regarding gender, occupational level (mean m: 2.74 vs. w. 2.66, p = < 0.001) and influence at work (mean m: 2.22 vs. w. 2.01, p = < 0.001) were higher among men than among women, whereas quality of leadership (mean w. 2.30 vs. m. 2.21, p = < 0.001) and mental distress (mean w. 23.7 vs. m. 19.5, p = < 0.001) was higher among women). Age did not differ significantly (mean m: 45.4 vs. w. 45.3, *p* = 0.469).

### Statistical analysis

#### Associations between the quality of leadership scale and mental distress

In preparation for the analysis, various probability distributions were fitted to the outcome variable (see Supplementary Table 2 online). The results showed that a normal distribution (linear model) provided a poor fit for both countries. The reason was an over-frequency of zero values (in relation to the distribution assumed for the values greater than zero) in both countries. Leaving out these zeros resulted in the best fit for a gamma distribution. One of several possible models for this data configuration is the two-part model^45,46^. For a two-part model, it is necessary to convert the outcome variable into two new variables. The first is a dichotomous variable that contains the values “0” and values greater than zero. The second variable is metric and contains all values greater than zero. The likelihood function on which the model is based can be maximized separately for each distribution component. This allows known statistical methods to be used for parameter estimation, a binomial model and a gamma model.

Thus, the chosen two-part model consists of logistic and generalized linear models that complement each other. The logistic regression was performed in the full cohorts, whereas the generalized linear model with a gamma link function was conducted only among those participants who had values of mental distress above 0 at follow-up. In the logistic regression, mental distress at follow-up was treated as a dichotomous measure. In the generalized linear model, mental distress at follow-up was treated as a continuous variable.

In both regression parts, associations between mental distress at follow-up and quality of leadership at baseline were estimated adjusted for baseline gender, age (a linear plus a quadratic term), mode of data collection and survey year (only in the Danish population), occupational level (categorical), influence at work and mental distress, treated as a dichotomous variable in the logistic regressions and as a continuous variable in the generalized linear models.

In the Danish cohort, the regression models assumed correlations between observations to belong to the same person. Due to deviating distributions of mental distress in the two cohorts, analyses were conducted separately. First, a logistic regression model was used to model all zero and non-zero values of the mental distress score, which was therefore treated as a dichotomous variable (see the coding of the mental distress score in the variable description above). Here, all observations (2,432 in the German cohort and 7,181 in the Danish cohort) were included in the analysis. Second, conditionally on all non-zero values, Generalized Estimating Equations (GEEs) with a gamma distribution and log-link-function were used to model the non-zero values, treating mental distress as a continuous variable (see the coding of the mental distress score in the variable description above). Therefore, only observations with non-zero values were included (2,318 in the German cohort, corresponding to 95% of all observations; 6,403 in the Danish cohort; corresponding to 89% of all observations) in the second analysis.

We also examined if the two-part model results differed between the two cohorts. To compare the regression coefficients for the associations of quality of leadership with mental distress obtained from the logistic and generalized linear models, for each part of the models were calculated the heterogeneity of the relative risk across the two cohorts by means of the Cochran Q test^47^.

For a sensitivity analysis only in the Danish cohort we repeated the two-part model analysis using a mental distress score based on the 5 mental health items from SF-36.

#### Software used

The analyses were conducted using SPSS 24 (GENLIN command) and Stata 18 (metan command).

### Ethics declarations

The S-MGA study was approved by the ethics commission of the Federal Institute of Occupational Safety and Health, approval number 006_2016_Müller. All employees in the sample were contacted by mail and the interviews were only conducted after each respondent gave their informed oral consent^32^. A written consent was given for the willingness to participate at follow-up. The DWECS has been notified to and registered by the Danish Data Protection Agency (Datatilsynet; see www.datatilsynet.dk/english for details). Questionnaire based studies do not need approval from the Danish National Committee on Biomedical Research Ethics (Den Centrale Videnskabetiske komité; see https://researchethics.dk/ for details).

## Results

### Occurrence of mental distress at follow-up in the whole cohorts

As the first part of the two-part model, we analysed – in the whole population being employees at baseline – associations in a logistic regression. Table 4 shows the results of the logistic regressions examining the associations between quality of leadership at baseline and the occurrence of mental distress at follow-up adjusted for mental distress and the other covariates at baseline. Higher quality of leadership was associated with a lower occurrence of mental distress at follow-up in both the German (Odds Ratio [OR] = 0.68, 95% confidence interval [CI] = 0.54;0.85), and the Danish cohort (OR = 0.88, 95% CI = 0.78;1.00; *p* = 0.032). The difference in the associations between the two countries was statistically significant (*p* = 0.036).

**Table 4:** Association between baseline quality of leadership and occurrence of mental distress as a dichotomous measure at follow-up five years later among employees in Germany and Denmark. 9,613 observations among 8,029 employees. Multiple logistic regressions. OR.

	Mental distress at follow-up as a dichotomous measure (MH-2 score 0 ‘No mental distress ‘, MH-2 = > 1 ‘Mental distress‘ ^a^)				
	Germany (*N* = 2,432; 2,432 observations)		Denmark (*N* = 5,587; 7,181 observations)		Difference Denmark-Germany
Quality of leadership ^b^	OR ^c^	95% CI ^d^	OR ^c^	95% CI ^d^	p ^e^
	0.68	0.54;0.85	0.88	0.78 ;1.00	0.036

^a^ Two item Mental Health scale (MH-2) from the 12 item Short Form Health Survey (SF-12)^35,36^.

^b^ Scale ranging from 0 to 4 from the Copenhagen Psychosocial Questionnaire (COPSOQ)^38,39^.

^c^ Odds Ratio (OR) adjusted for the baseline variables gender, age, mode of data collection (only Denmark), survey year (only Denmark), occupational level, influence at work and mental distress as a dichotomous measure.

^d^ 95% Confidence interval (CI), two-tailed.

^e^ p-value for heterogeneity of OR across the two cohorts through the Cochran Q test^47^.

In a sensitivity analysis, we repeated the logistic regression in the Danish cohort using a mental distress measure based on all 5 mh-5 items from SF-36. Here, higher quality of leadership was not associated with a lower occurrence of mental distress (OR = 0.91, 95% CI = 0.74;1.12).

Regarding covariates, in the German cohort, baseline occupational level and mental distress were associated with mental distress at follow-up. In the Danish cohort, the baseline covariates year of data collection, gender, age (squared), occupational level, mental distress and influence at work were associated with mental distress at follow-up (Data not shown).

### Extent of mental distress at follow-up

As the second part of the two-part model, we analysed – among those in the employee population who had mental distress (> 0) at follow-up – associations in a generalized linear model. Table 5 shows the results of the generalized linear models examining the associations between quality of leadership at baseline and the extent of mental distress as a continuous measure at follow-up adjusted for mental distress (continuous score) and covariates at baseline. Higher quality of leadership was associated with a decreased level of mental distress at follow-up in the Danish cohort [Exp(β) = 0.96, 95% CI = 0.94;0.98], but not in the German cohort [Exp(β) = 1.00, 95% CI = 0.97;1.02]. The difference in the associations between the two countries was statistically significant (*p* = 0.014).

**Table 5:** Association between baseline quality of leadership and the extent of mental distress as a continuous measure at follow-up five years later among employees in Germany and Denmark with mental distress distress at follow-up (MH-2 score > 0). 8,969 observations among 7,587 employees. Multiple generalized linear models. Exp(β).

	Mental distress (MH-2) ^a^ at follow-up as a continuous measure				
	Germany (*N* = 2,306; 2,306 observations)		Denmark (*N* = 5,281; 6,663 observations)		Difference Denmark-Germany
Quality of leadership ^b^	Exp(β) ^c^	95% CI ^d^	Exp(β) ^c^	95% CI ^d^	p ^e^
	1.00	0.97;1.02	0.96	0.94;0.98	0.014

^a^ Two item Mental Halth (MH-2) scale from the 12 item Short Form Health Survey (SF-12)^35,36^.

^b^ Scale ranging from 0 to 4 from the Copenhagen Psychosocial Questionnaire (COPSOQ)^38,39^.

^c^ Exp(β) adjusted for the baseline variables gender, age, mode of data collection (only Denmark), survey year (only Denmark), occupational level, influence at work and mental distress as a continuous measure.

^d^ 95% Confidence interval (CI), two tailed.

^e^ p-value for heterogeneity of Exp(β) across the two cohorts through the Cochran Q test^47^.

In a sensitivity analysis, we repeated the generalized linear model in the Danish cohort using a mental distress measure based on all 5 mh-5 items from SF-36. Also in this analysis, higher quality of leadership was associated with a decreased level of mental distress at follow-up (OR = 0.98, 95% CI = 0.96;1.00; *p* = 0.023).

Regarding covariates, in the German cohort, baseline gender, age (linear), age (squared) and mental distress were associated with mental distress at follow-up. In the Danish cohort, the baseline covariates year of data collection, mode of data collection, gender, age (squared), mental distress and influence at work were associated with mental distress at follow-up (Data not shown).

## Discussion

The present study suggests that quality of leadership is linked to reduced mental distress in both Danish and German working populations. In Germany, and to a lesser degree Denmark, baseline quality of leadership appears to operate as a risk factor for the occurrence of mental distress. In Denmark, but not in Germany, baseline quality of leadership seems to be associated with the subsequent extent of mental distress.

The hypothesis that the association between quality of leadership and mental distress would be stronger in Germany than in Denmark was not consistently corroborated due to these contradictory results. In terms of the occurrence of mental distress, the association between higher quality of leadership at baseline and a reduced risk of mental distress at follow-up was stronger in Germany than in Denmark. However, regarding the extent of mental distress, the association between higher quality of leadership and lower levels of mental distress at follow-up was stronger in Denmark than in Germany, which points against our hypothesis.

The results indicate that the relationship between quality of leadership and mental distress is more complex than initially anticipated. The likelihood of developing mental distress seems to differ from the likelihood of worsening mental distress among those already affected. These findings underscore the importance of analysing measurements of mental distress in relatively healthy populations, where the distribution of mental distress is skewed.

### Comparison with other studies

As stated in the introduction, four longitudinal studies from Scandinavia have previously investigated if quality of leadership was associated with mental distress^14,18–20^. Three studies, two on heterogeneous Norwegian and Danish samples of the working population and one on eldercare workers in Denmark, showed that higher quality of leadership was associated with subsequent mental or depressive disorder^14,18,20^. The findings of the Norwegian study and the Danish study on eldercare workers cannot be compared to the present study. This is due to the more liberal cut-off points for mental distress used in the dichotomous measure applied in the current study compared to those in the other two studies. Also, the outcome of the Danish study conducted on a heterogenous sample differed from the present study, as the former employed medication and hospitalizations for depression and anxiety as outcome measures. In the fourth study, which analysed nationwide samples of the working population in Sweden and Denmark, no association was found^19^. Madsen et al. (2014) used the same 2005 baseline data for Denmark as employed in the present study for the Danish cohort. However, their study differed by utilizing a different indicator of mental distress, specifically the purchase of antidepressants. In addition, four longitudinal studies have investigated specific beneficial leadership styles as risk factors for mental distress^14,21–23^. They reported mixed findings on fair^14,21^ and transformational leadership^22,23^, while consistent findings were observed for transformational leadership^14,21^. However, each of these leadership styles was only investigated by two studies each. In all the aforementioned studies, the significant associations were found between low quality of leadership and higher level of mental distress. Therefore, while there are indications that quality of leadership – or specific beneficial leadership styles - is associated with the subsequent development of mental distress, the limited number of studies, the predominant focus on Scandinavian countries, and methodological differences in measurements and definitions make it difficult to draw definitive conclusions.

### Study strengths and limitations

First, it is a strength of the present study that it is based on random samples of the German and Danish working populations. Second, the study’s prospective design allows for a stronger assessment of potential causation, as the risk factor (quality of leadership) was measured prior to the outcome (mental distress)^48,49^.

The strengths of this study must be balanced against some limitations. First, this is an observational study in which exposure to quality of leadership was not randomly assigned. Second, in the German cohort, attrition was high and was related to age and occupational level but not to gender, quality of leadership, or mental distress (Tables 1 and 2). In the Danish cohort, attrition was lower and was related to gender, age and occupational level, as well as, to some extent, mental distress, but not to quality of leadership (Tables 1 and 2). These biases would lead to an underrepresentation of, for example, individuals in lower occupational level, thus leading to less precise risk estimates. We cannot rule out the possibility that individuals with high levels of mental distress at follow-up may have opted out of the study, potentially causing an underestimation of associations between quality of leadership and mental distress. Third, we measured mental distress with the SF-12, which only included two of the original five measures mental distress (i.e. MH-5) included in the SF-36. This may have resulted in a less reliable measure of mental distress and potentially led to less precise estimates of associations in the present study. We are not aware of other studies using only these two items. We repeated the analysis in the Danish cohort using all 5 items on mental health from SF-36. Results showed the same pattern as when only using the two item measure (see results section). Fourth, the measure of quality of leadership was not theory based; it rather describes overall aspects leadership and not specific leadership styles^10^. Quality of leadership can be seen as the leaders’ capacity to help subordinates in fulfilling a goal by means of processes of influence^6^. Fifth, even if the present study employed validated measures of quality of leadership^38,39^, we cannot rule out reporting bias among those with mental distress at baseline. Sixth, a comparison between only two countries is insufficient to establish whether differences in the associations can be attributed to welfare state characteristics of workplace culture indicators^27,28^. Seventh, we compared two different time windows: the Danish data covers the period from 2000 to 2010, while the German data spans from 2012 to 2017. One should also take into account that national welfare state characteristics and workplace culture indicators change over time, which may also affect quality of leadership and its effect on mental health^28,50^. Eighth, the follow-up period of 5 years is relatively long, which might have overlooked shorter-term effects^51^. This might have led to conservative estimates of associations between quality of leadership and mental distress. Ninth, the number of observations was significantly smaller in the German cohort (*N* = 2484) compared to the Danish cohort (*n* = 7181), potentially leading to greater variance in the German cohort. However, the study was able to identify differences in the country-specific estimates in both logistic and generalized linear models, indicating that the findings were not constrained by limited statistical power.

### Perspectives

From a practical perspective, the results suggest that improving quality of leadership might lead to reduced mental distress beyond a Scandinavian context. Research from other non-Scandinavian national contexts is needed to gather more information on the relationship between quality of leadership and mental distress, which could help guide future intervention studies.

From a research perspective, we suggest that quality of leadership is investigated as possible risk factor for mental distress in further cross-national prospective studies. In the present paper, we have, for the first time, investigated the prospective impact of quality of leadership in a non-Scandinavian country and identified an association. However, the question remains whether quality of leadership is associated with mental distress in countries beyond the Scandinavian context or Germany. Additionally, the study indicates that associations between quality of leadership and mental distress vary depending on how mental distress is measured—whether as a dichotomous or continuous variable. We also recommend reanalysing existing data to shed further light onto this matter.

## Electronic supplementary material

Below is the link to the electronic supplementary material.

Supplementary Material 1

## Data Availability

A scientific use file (SUF) containing both the 2012 wave and the 2017 wave of the cohort is available at the Research Data Centre of the Federal Institute of Occupational Safety and Health (doi: 10.48697/smga.w1w2.suf.).
