# Correction: A comparison among employees in Germany and Denmark of associations between quality of leadership and subsequent 5-year development of mental distress

**DOI:** 10.1038/s41598-025-30371-0

**Published:** 2025-12-03

**Authors:** Hermann Burr, Norbert Kersten, Kathrine Sørensen, Jeppe K. Sørensen, Louise Dalsager, Ida E. H. Madsen, Ina Schöllgen, Angelo d’Errico, Uwe Rose, Reiner Rugulies

**Affiliations:** 1 Department of Work and Health, Federal Institute for Occupational Safety and Health (BAuA), 10317 Berlin, Germany; 2 National Research Centre for the Working Environment (NRCWE), 2100 Copenhagen Ø, Denmark; 3 Department of Psychology, University of Copenhagen, 1353 Copenhagen K, Denmark; 4 National Institute of Public Health, University of Southern Denmark, 1455 Copenhagen K, Denmark; 5 Department of Epidemiology, Local Health Unit TO 3, 10095 Grugliasco Turin, Italy; 6 Section of Epidemiology, Department of Public Health, University of Copenhagen, 1353 Copenhagen K, Denmark

Correction to: *Scientific Reports* 10.1038/s41598-025-92650-0, published online 06 March 2025

The original version of this Article contained a repeated error, where the number of observations for the Danish employee cohort and for the German employee cohort were incorrect. The calculations relating to these observations have been corrected accordingly.

As a result, in the Methods section, under the subheading ‘Danish employee cohort’,

“We extracted the 2000–2005 and 2005–2010 cohorts from DWECS, which had a responses of 76% and 65% respectively at baseline and a responses of 72% and 65% respectively at follow-up (Table 3). DWECS was an open population-based cohort study investigating work and health among the Danish workforce through repeated questionnaire assessments conducted every five years^33,34^. Thus, a DWECS participant could have been included in one or both 5-year cohorts. The cohort comprised 7,818 observations from 5,597 people who were employed at baseline, took part in at least one follow-up, and had non-missing information on all key variables (Fig. 1). Respondents either took part in computer assisted telephone interviews or filled out paper questionnaires. The change in mode of data collection from telephone interviews was partly introduced in 2005 and was fully implemented in 2010. Among the 7,818 observations, 790 were collected through telephone interviews at both baseline and follow-up, 3572 were collected through questionnaires at both baseline and follow-up, and 2819 observations were via telephone interviews at baseline and via questionnaire at follow-up.”

now reads:

“We extracted the 2000–2005 and 2005–2010 cohorts from DWECS, which had a responses of 76% and 65% respectively at baseline and a responses of 72% and 67% respectively at follow-up (Table 3). DWECS was an open population-based cohort study investigating work and health among the Danish workforce through repeated questionnaire assessments conducted every five years^33,34^. Thus, a DWECS participant could have been included in one or both 5-year cohorts. The cohort comprised 7,181 observations from 5,597 people who were employed at baseline, took part in at least one follow-up, and had non-missing information on all key variables (Fig. 1). Respondents either took part in computer assisted telephone interviews or filled out paper questionnaires. The change in mode of data collection from telephone interviews was partly introduced in 2005 and was fully implemented in 2010. Among the 7,818 observations, 790 were collected through telephone interviews at both baseline and follow-up, 3,572 were collected through questionnaires at both baseline and follow-up, and 2,819 observations were via telephone interviews at baseline and via questionnaire at follow-up.”

In the Variables section, under the subheading ‘Correlations between the independent variables’,

“Regarding gender, influence at work (mean m: 1.83 vs. w. 1.56, p = < 0.001) was higher among men than among women, whereas quality of leadership (mean w. 2.31 vs. m. 2.22, p = < 0.001) and mental distress (mean w. 39.1 vs. m. 34.6, p = < 0.001) was higher among women). Age (mean m: 45.4 vs. w. 45.3, *p* = 0.126) and occupational level (mean w. 47.1 vs. 46.7, *p* = 0.662) did not differ significantly.”

now reads:

“Regarding gender, influence at work (mean m: 1.83 vs. w. 1.56, p = < 0.001) was higher among men than among women, whereas quality of leadership (mean w. 2.31 vs. m. 2.22, p = < 0.023) and mental distress (mean w. 39.1 vs. m. 34.6, p = < 0.001) was higher among women). Age (mean m: 46.7 vs. w. 47.1, *p* = 0.126) and occupational level (mean w. 2.7 vs. m. 2.7, *p* = 0.662) did not differ significantly.”

Under the subheading ‘Associations between the quality of leadership scale and mental distress’,

“Therefore, only observations with non-zero values were included (2,318 in the German cohort, corresponding to 94% of all observations; 6,403 in the Danish cohort; corresponding to 93% of all observations) in the second analysis.”

now reads:

“Therefore, only observations with non-zero values were included (2,318 in the German cohort, corresponding to 95% of all observations; 6,403 in the Danish cohort; corresponding to 89% of all observations) in the second analysis.”

Finally, in the Discussion section, under the subheading ‘Study strengths and limitations’,

“Ninth, the number of observations was significantly smaller in the German cohort (*N* = 2,432) compared to the Danish cohort (*n* = 5,597), potentially leading to greater variance in the German cohort.”

now reads:

“Ninth, the number of observations was significantly smaller in the German cohort (*N* = 2,484) compared to the Danish cohort (*n* = 7,181), potentially leading to greater variance in the German cohort.”

The original Article has been corrected.

