# Supplementary material for: A comparison among employees in Germany and Denmark of associations between quality of leadership and subsequent 5-year development of mental distress
Source: Sci Rep. 2025 Mar 6;15:7802. doi: 10.1038/s41598-025-92650-0 (PMC11885610; doi:10.1038/s41598-025-92650-0)
Supplement: Supplementary file 1 — Supplementary Material 1 [file 41598_2025_92650_MOESM1_ESM.docx]

## Supplementary Table S1. Description of the analysed population distinguishing between mode of data collection. 9,613 observations among 8,029 employees

|  | Employees in Germany | | | | | | | Employees in Denmark | | | | | | | | | | | | | |
| --- | --- | --- | --- | --- | --- | --- | --- | --- | --- | --- | --- | --- | --- | --- | --- | --- | --- | --- | --- | --- | --- |
|  | N | % | Mean | Std. dev. | Skewness | α ^a^ | IICR ^b^ | N, observations | % | Mean | | | Std. dev. | | | Skewness | | α ^a^ | | IICR ^b^ | |
|  |  |  | Int. | Int. | Int. | Int. | Int. |  |  | Int. | Quest. | | Int. | Quest. | | Int. | Quest. | Int. | Quest. | Int. | Quest. |
| Gender, Women; % |  |  |  |  |  |  |  |  |  |  |  | |  |  | |  |  |  |  |  |  |
| Men | 1,189 | 49 |  |  |  |  |  | 3,273 | 46 |  |  | |  |  | |  |  |  |  |  |  |
| Women | 1,243 | 51 |  |  |  |  |  | 3,908 | 54 |  |  | |  |  | |  |  |  |  |  |  |
| Age, baseline (31-60) |  |  | 46.9 | 7.6 | -.2 |  |  |  |  | 44.7 | | 46.0 | 8.2 | | 8.1 | 0.1 | 0.0 |  |  |  |  |
| 31-40 years | 548 | 23 |  |  |  |  |  | 2,332 | 32 |  |  | |  |  | |  |  |  |  |  |  |
| 41-50 years | 1,028 | 42 |  |  |  |  |  | 2,623 | 37 |  |  | |  |  | |  |  |  |  |  |  |
| 51-60 years | 856 | 35 |  |  |  |  |  | 2,226 | 31 |  |  | |  |  | |  |  |  |  |  |  |
| Occupational level, baseline |  |  |  |  |  |  |  |  |  |  |  | |  |  | |  |  |  |  |  |  |
| Unskilled workers (ISCO group 9) c | 137 | 6 |  |  |  |  |  | 655 | 9 |  |  | |  |  | |  |  |  |  |  |  |
| Skilled workers (ISCO group 4-8) c | 1,022 | 42 |  |  |  |  |  | 2,744 | 38 |  |  | |  |  | |  |  |  |  |  |  |
| Semi-professionals (ISCO gr. 3) c | 682 | 28 |  |  |  |  |  | 1,891 | 26 |  |  | |  |  | |  |  |  |  |  |  |
| Professionals/managers (ISCO group 1,2) c | 591 | 24 |  |  |  |  |  | 1,891 | 26 |  |  | |  |  | |  |  |  |  |  |  |
| Mode of data collection |  |  |  |  |  |  |  |  |  |  |  | |  |  | |  |  |  |  |  |  |
| Interview baseline & follow-up | 2,432 | 100 |  |  |  |  |  | 790 | 11 |  |  | |  |  | |  |  |  |  |  |  |
| Interview baseline & quest. follow-up | 0 | 0 |  |  |  |  |  | 2,819 | 39 |  |  | |  |  | |  |  |  |  |  |  |
| Questionnaire baseline & follow-up | 0 | 0 |  |  |  |  |  | 3,572 | 50 |  |  | |  |  | |  |  |  |  |  |  |
| Quality of leadership, baseline (0-4) |  |  | 2.3 | 0.9 | -0.4 | 0.84 | 0.52-0.65 |  |  | 2.4 | 2.1 | | 0.8 | 0.9 | | -0.5 | -0.3 | 0.84 | 0.90 | 0.50-0.63 | 0.63-0.74 |
| Influence at work, baseline (0-4) |  |  | 1.7 | 1.0 | 0.2 | 0.71 | 0.32-43 |  |  | 2.0 | 2.2 | | 1.1 | 0.9 | | -0.1 | -0.,2 | 0.73 | 0.82 | 0.34-0.51 | 0.49-0.60 |
| Mental distress, baseline (0-4) |  |  | 36.9 | 20.1 | 0.3 | 0.73 | 0.58 ^d^ |  |  | 18.3 | 26.1 | | 15.1 | 18.5 | | 1.6 | 0.9 | 0.62 | 0.60 | 0.45 | 0.42 |

^a^ Cronbach’s α.

^b^ Inter item correlation range.

^c^ Occupational level was largely based on the four category skill level International Standard Classification of Education (ISCED) , see method section ^1,2^. ISCED uses a categorization of the International Standard Classification of Occupations (ISCO) ^2^.

^d^. As the scale was based on only two items, there was only one inter item correlation.

## Suppelmentary Table S2. Quasi-likelihood-values of a preliminary analysis of the fit of a normal distribution to the outcome in comparison with a gamma distribution fitted to the positive outcome values

|  | Employees in Germany | Employees in Denmark |
| --- | --- | --- |
| Normal distribution (linear model) | 102261.5 | 64759.6 |
| Gamma distribution | 574.3 | 2146.0 |

See also first paragraph under the subheading ‘Statistical analyses’ on page 6.

# References

1 Hagen, F. *Levels of Education: Relation between ISCO Skill Level and ISCED Categories*, <<http://www.fernunihagen.de/FTB/telemate/database/isced.htm#ISCO>> (2015).

2 International Labor Office Staff. *International Standard Classification of Occupations 2008 (ISCO-08): Structure, Group Definitions and Correspondence Tables*. (International Labour Office, 2012).
